# Inconvenience due to travelers' diarrhea: a prospective follow-up study

**DOI:** 10.1186/1471-2334-11-322

**Published:** 2011-11-20

**Authors:** Darius Soonawala, Jessica A Vlot, Leo G Visser

**Affiliations:** 1 Department of Infectious and Tropical Diseases, Leiden University Medical Center, Leiden, The Netherlands

## Abstract

**Background:**

Limited data exist documenting the degree to which travelers are inconvenienced by travelers' diarrhea (TD). We performed a prospective follow-up study at the travel clinic of Leiden University Medical Center in The Netherlands to determine the degree of inconvenience and to determine how experiencing TD affects travelers' perception.

**Methods:**

Healthy adults who intended to travel to the (sub)tropics for less than two months were invited to take part. Participants filled out a web-based questionnaire before departure and after returning home. TD was defined as three or more unformed stools during a 24-hour period.

**Results:**

390 of 776 Eligible travelers completed both questionnaires. Participants' median age was 31 years and mean travel duration 23 days. Of 160 travelers who contracted TD (incidence proportion 41%, median duration of TD episode 2.5 days) the majority (107/160, 67%) could conduct their activity program as planned despite having diarrhea. However, 21% (33/160) were forced to alter their program and an additional 13% (20/160) were confined to their accommodation for one or more daylight days; 53 travelers (33%) used loperamide and 14 (9%) an antibiotic. Eight travelers (5%) consulted a physician for the diarrheal illness. When asked about the degree of inconvenience brought on by the diarrheal illness, 39% categorized it as minor or none at all, 34% as moderate and 27% as large or severe. In those who regarded the episode of TD a major inconvenience, severity of symptoms was greater and use of treatment and necessity to alter the activity program were more common. Travelers who contracted travelers' diarrhea considered it less of a problem in retrospect than they had thought it would be before departure.

**Conclusion:**

Conventional definitions of TD encompass many mild cases of TD (in our study at least a third of all cases) for which treatment is unlikely to provide a significant health benefit. By measuring the degree of inconvenience brought on by TD, researchers and policy makers may be able to better distinguish 'significant TD' from mild TD, thus allowing for a more precise estimation of the size of the target population for vaccination or stand-by antibiotic prescription and of the benefit of such measures.

## Background

Travelers' diarrhea (TD) affects 20-50% of travelers from industrialized regions to developing countries [1-3]. Many travel medicine experts recommend loperamide for mild TD and self-administered antibiotic treatment in case of moderate or severe TD [4-6]. Compared with placebo, antibiotics shorten the duration of diarrhea by 0.7-1.5 days and reduce the number of unformed stools per 24 hour time interval by 1.6 on the first day of treatment, 2.1 on the second day, and 1.4 on the third day [7]. No studies exist that have assessed to what extent early antibiotic treatment significantly impacts the subjective and objective (i.e. incapacitation) degree of inconvenience due to TD. The benefit of prescribing all travelers with antibiotics for self-treatment in case of TD should be weighed against the drawbacks. Although side-effects are seldom serious, use of an antibiotic makes a person more susceptible to colonization by drug resistant *Enterobacteriaceae *[8,9]. Furthermore, large-scale use and disposal of antibiotics in the environment induces resistance among pathogens. For these reasons, there are pro- and opponents regarding routine pre-travel prescription of stand-by antibiotics for travelers [10]. An argument favoring routine prescription is that there is an increasing concern about purchasing antibiotics abroad, many being false. A central argument for those who advocate wide-spread use of antibiotics for TD is that it can cause considerable inconvenience, ruin holidays and cause financial loss and that it may cause chronic gastro-intestinal complaints [6,11,12]. A number of studies describe the impact of TD on quality of life and incapacitation [2,3,13-15]. Of those with TD, 20-45% is unable to pursue planned activities for 1 day and the quality of life is affected, mostly with regard to the ability to participate in leisure activities, sexual activity, and the feeling of general well-being [13]. The present prospective follow-up study was designed to determine the degree of subjective and objective inconvenience that Dutch travelers experience when they contract diarrhea during travel to the (sub)tropics. In addition we determined how an episode of TD affects travelers' perception of TD and we explored risk factors.

## Methods

### Design and study population

This was a single-center prospective cohort study at the travel clinic of Leiden University Medical Center in The Netherlands. It was conducted from March until November 2010. Healthy adults who visited the travel clinic and intended to travel to the (sub)tropics were invited to take part by way of an informative letter. The letter was attached to a standard intake form that clients fill out before their appointment at the travel clinic. All who read the letter were asked to fill out an accompanying answer card that provided three options: (i) "yes, I want to participate", (ii) "no, I do not want to participate", (iii) "I am not eligible to participate". Exclusion criteria were: travel duration of more than two months, use of systemic immunosuppressive medication, history of inflammatory bowel disease or insulin-dependent diabetes mellitus. Participants were sent two web-based questionnaires via e-mail, the first before departure, and the second a week after returning home. In The Netherlands no formal approval by a medical ethics committee is required for this kind of questionnaire study.

The pre-travel consult was not different for participants than for other travelers. All received a brochure about preventive measures and self-treatment with loperamide and oral rehydration solution in case of TD. In The Netherlands, pre-travel supply of antibiotics for self-treatment in case of TD is restricted to high-risk travelers who are at increased risk of severe infection or dehydration, and to those who travel to remote areas with limited access to health care facilities [16].

### Definition of travelers' diarrhea

In order to avoid misinterpretation we used a straightforward definition of TD. In the questionnaires TD was defined as: the passage of three or more unformed stools during a 24-hour period with or without additional symptoms [14,17]. In the analyses, 'classic TD' was defined separately as: the passage of three or more unformed stools during a 24-hour period with one or more symptoms of enteric disease such as nausea, abdominal cramps, vomiting, fever or fecal urgency [18,19].

### Questionnaires

The first questionnaire (Q1) consisted of questions on past travel to the tropics, past experience with TD and past inconvenience due to TD. In addition, we surveyed the incidence of diarrhea among participants during a two-month period in The Netherlands and during past travel to the tropics. The second questionnaire (Q2) was sent within a week after returning home and dealt with travel characteristics, the incidence of TD and accompanying symptoms, the use of anti- diarrheal medication, the incidence of other health problems, the incidence of TD among travel companions, health-care use for TD and subjective and objective inconvenience due to TD. The *objective *degree to which TD inconvenienced travelers was measured by asking: "To what extent were you inconvenienced by your episode of diarrhea?". Participants could choose one of the following answers: (i) "I interrupted my journey and returned home due to the diarrhea and abdominal complaints", (ii) "I was ill, I altered my activity program and stayed indoors for one or more days due to the diarrhea and abdominal complaints", (iii) "I altered my activity program due to the diarrhea and abdominal complaints", or (iv) "despite the episode of diarrhea, I could take part in all planned activities". Some travelers may have had more than one episode of TD. All questions concerning symptoms of TD and the degree of inconvenience due to TD pertained to the most severe episode. The *subjective *degree of inconvenience due to TD was measured by asking: "To what degree did you experience inconvenience due to the episode of diarrhea?". Participants could choose from the following answers: (i) "no inconvenience", (ii) "a minor degree of inconvenience", (iii) "a moderate degree of inconvenience", (iv) "a large degree of inconvenience", or (v) "a severe degree of inconvenience". In addition, we explored how an episode of TD during travel changed travelers' own perception of TD. This was done as follows. Before departure we asked: "If you were to contract travelers' diarrhea with fecal urgency and abdominal cramps for three days, how large a problem would you consider this to be?". (i) "no problem", (ii) "a small problem", (iii) "neither a small nor a large problem", (iv) "a large problem", (v) "a very large problem". After returning home all travelers were presented a similar scenario pertaining to a hypothetical future travel. We thought that the answer to this question would change in travelers who had contracted TD and would remain the same in those who had not. The overall direction in which the answer changes, reflects how experiencing an episode of TD influences the perception of TD. We piloted the questionnaire among travelers, acquaintances and staff of the department of Clinical Epidemiology at Leiden University Medical Center.

### Data editing

Travel destination was categorized according to the United Nations (UN) International Migrant Stock [20]. Travel destination was also categorized according to the UN Human Development Index (HDI) value (0 to 1) and UN HDI category (high, medium, low) [21]. The HDI is based upon indicators of life expectancy, education and living standards. If a participant visited more than one country, the HDI value of the country with the lowest HDI was used. In regression analyses, continuous variables that were not linearly associated with the dependent variable were categorized based on exploratory analyses of the continuous data in small categories to see at which values of the continuous variable the regression coefficient changed.

### Sample size

The sample size was based on the rule of thumb that 10 cases are needed for each covariate that is introduced in a logistic regression model [22]. Based on an assumed incidence proportion (i.e. the incident number of cases in relation to the size of the population) for TD of 25% we estimated that 400 travelers were needed to be able to introduce a maximum of 10 separate covariates in a logistic regression analysis.

### Regression analyses

In a prediction model we explored which variables significantly increased the odds of contracting classic TD. Categorical variables were analyzed with χ^2^-tests and continuous variables with t-tests. Variables with *p *< 0.2 were entered in a multiple logistic regression model based on maximum likelihood estimation. Interaction terms were not entered in the model to prevent overfitting and because interaction was deemed unlikely. Cook's distance values, leverage values and standardized residuals were examined to detect cases that might be influencing the model disproportionately. Variance inflation factors were examined to test whether any covariates were highly collinear. The relative strength of each covariate in the final regression model was determined by computing the delta in Nagelkerke R^2 ^when one covariate was deleted and by dividing delta by the final model's Nagelkerke R^2^. In another logistic regression analysis restricted to travelers who had TD, we explored which person- and travel characteristics predicted incapacitation due to TD. In a third model we explored which symptoms predicted incapacitation due to TD. All analyses were done using PASW Statistics, version 18.0, IBM^®^. Statistical significance was defined as a *p*-value < 0.05.

## Results

### Study population and travel characteristics

**Figure 1:**
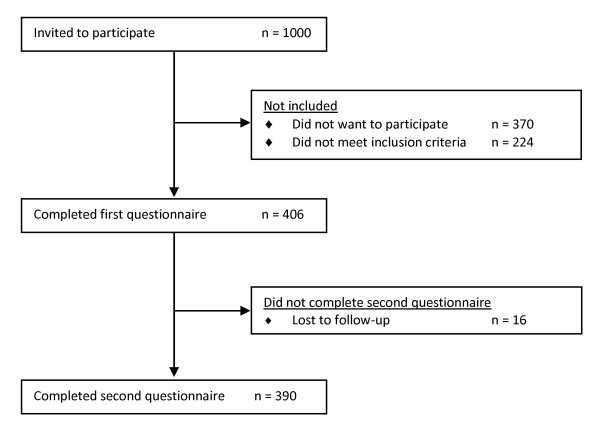
**Flowchart of participants in the study of inconvenience due to travelers' diarrhea**.

At our travel clinic 776 of 1,000 travelers fulfilled the inclusion criteria of which 406 provided informed consent (response rate 52%). Of the 224 people who were not eligible to participate, travel duration in excess of two months was the most common exclusion criterion. Three hundred and ninety travelers completed both the pre- and post- travel questionnaire (follow-up rate 96%) (Figure 1). The median age was 31 years (IQR 24-50 years). The majority was female (65%), and had completed higher education (62%) (i.e. a Bachelor degree). Person- and travel characteristics are described in Table 1 and 2. Tourism was the main reason for travel and South-eastern Asia was visited most frequently (31%). The mean travel duration was 23 days (range 4-57 days). At the pre-travel consult, 27 travelers (7%) received a stand-by antibiotic prescription (ciprofloxacin or azitromycin). In total 335 travelers (86%) carried treatment for TD in their travel-kit, mainly loperamide (282/390, 72%), oral rehydration solution (229/390, 59%) or activated carbon (83/390, 21%).

**Table 1:** Travel characteristics and risk factors for travelers' diarrhea in a cohort of 390 Dutch travelers.

Characteristic	All n = 390	Classic TD n = 144	No TD n = 246	Univariate OR [95% CI]	*p*-value	Multivariate OR [95% CI]	Relative contribution of each characteristic to the model's R^2 ^(%)
**Gender, female**^1^	253 (65)	98 (68)	155 (63)	1.25 [0.81-1.93]	0.31		
**Age**					< 0.001		18.6
18-34 years	217 (56)	102 (71)	115 (47)	1.0		1.0	
≥ 35 years	173 (44)	42 (29)	131 (53)	0.36 [0.23-0.56]		0.38 [0.23-0.65]	
**Use of an antacid**^2^	24 (6)	14 (10)	10 (4)	2.54 [1.10-5.88]	0.03	2.95 [1.14-7.60]	6.8
**Born in the tropics**^3^	19 (5)	5 (3)	14 (6)	0.60 [0.21-1.69]	0.33		
**Traveled to the tropics in the preceding 5 years**^4^	251 (64)	91 (63)	160 (65)	0.92 [0.60-1.42]	0.71		
**Mean travel duration **- *days *(SE)	22.9 (0.65)	25.6 (1.2)	21.4 (0.7)	1.03 [1.01-1.04]	0.002	1.02 [0.99-1.04]	2.7
**Travel destination, Human Development Index**					0.02		10.6
High	79 (20)	18 (13)	61 (25)	1.0		1.0	
Medium	219 (56)	90 (63)	129 (54)	2.36 [1.31-4.27]	0.004	2.29 [1.22-4.33]	
Low	92 (24)	36 (25)	56 (23)	2.18 [1.11-4.27]	0.02	2.51 [1.19-5.33]	
**Main travel purpose**^5^							
Holiday	236 (61)	89 (62)	147 (60)	1.09 [0.72-1.66]	0.69		
Visit friends/relatives	55 (14)	13 (9)	42 (17)	0.48 [0.25-0.93]	0.03	0.56 [0.27-1.19]	3.3
Business/professional	32 (8)	5 (4)	27 (11)	0.30 [0.11-0.81]	0.02	0.31 [0.11-0.88]	7.6
Study	51 (13)	29 (20)	22 (9)	2.57 [1.41-4.67]	0.002	1.16 [0.56-2.40]	0.2
Volunteer work	16 (4)	8 (6)	8 (3)	1.75 [0.64-4.77]	0.27		
**Type of travel**^6^							
Self-arranged, not backpacking	170 (44)	56 (39)	114 (46)	0.74 [0.49-1.12]	0.15	1.17 [0.68-2.01]	0.4
Backpacking	85 (22)	44 (31)	41 (17)	2.20 [1.35-3.58]	0.002	1.89 [0.96-3.70]	4.5
Organized group travel	108 (28)	37 (26)	71 (29)	0.85 [0.54-1.36]	0.50		
Other	27 (7)	7 (5)	20 (8)	0.58 [0.24-1.40]	0.23		
**Type of accommodation**^7^							
Luxury hotel only	98 (25)	43 (30)	55 (22)	1.48 [0.93-2.36]	0.10	2.94 [1.64-5.29]	18.2
Budget hotel only	95 (24)	34 (24)	61 (25)	0.94 [0.58-1.52]	0.79		
Camping (tent/camper)	26 (7)	9 (6)	17 (7)	0.90 [0.39-2.07]	0.80		
Holiday home	15 (4)	5 (4)	10 (4)	0.85 [0.28-2.53]	0.77		
Stayed with friends or relatives	12 (3)	3 (2)	9 (4)	0.56 [0.15-2.10]	0.39		
Stayed with locals	13 (3)	8 (6)	5 (2)	2.84 [0.91-8.84]	0.07	2.91 [0.80-10.55]	3.4
Combination of the above^†^	131 (34)	42 (29)	89 (36)	0.73 [0.47-1.13]	0.16		
Diarrheal episode 2 months prior to departure*					0.003		
No	195/251 (78)	61/91 (67)	134/160 (84)	1.0			
Yes	56/251 (22)	30/91 (33)	26/160 (16)	2.54 [1.38-4.65]			
Subjective susceptibility for travelers' diarrhea*					0.12		
Never	104/251 (41)	33/91 (36)	71/160 (44)	1.0			
Sometimes	121/251 (48)	44/91 (48)	77/160 (48)	1.23 [0.71-2.14]	0.47		
Often/Always	26/251(10)	14/91 (15)	12/160 (8)	2.51 [1.05-6.02]	0.04		

Data are presented as n (%), unless otherwise stated. OR: odds ratio; SE: standard error of the mean; CI: confidence interval. *P*-values based on χ^2^-tests for categorical variables and t-tests for continuous variables. Variables with *p *< 0.2 were included in the multivariate logistic regression model. Reference category: ^1^male gender, ^2^no use of an antacid, ^3^born in The Netherlands, ^4^not having traveled to the tropics in the preceding 5 years, ^5^not the specified travel purpose, ^6^not the specified type of travel, ^7^not having stayed in the specified type of accommodation. ^†^Not included in the multivariate model because it is not a uniform category. *Not included in the multivariate logistic regression model because subjects who had not traveled to the tropics in the past 5 years had missing values for these variables. Model: constant = 0.19, Nagelkerke's R^2 ^= 0.22, Hosmer and Lemeshow test for goodness of fit *p *= 0.4.

**Table 2:** Travelers' diarrhea, incidence proportions and incidence rates for 390 Dutch travelers.

Travel destination	Travelers - *n*	TD cases - *n*	TD incidence proportion - % (SE)	Mean travel duration - days	TD Incidence rate - per 100 pdt (SE)
Northern Africa	17	7	41 (12.3)	10.4	3.95 (1.47)
South-central Asia	31	16	52 (9.1)	20.2	2.55 (0.63)
Central America and Caribbean	24	11	46 (10.4)	18.9	2.42 (0.72)
South-eastern Asia	121	61	50 (4.6)	22.5	2.25 (0.28)
Eastern Africa	57	25	44 (6.6)	23.4	1.88 (0.37)
Central Africa	7	3	43 (20.2)	23.4	1.83 (1.05)
Central and Western Asia	32	8	25 (7.8)	14.3	1.75 (0.61)
Western Africa	15	7	47 (13.3)	28.6	1.63 (0.61)
Southern Africa	15	4	27 (11.8)	23.1	1.16 (0.58)
Eastern Asia	36	11	31 (7.8)	29.4	1.04 (0.31)
South America	46	7	15 (5.4)	26.4	0.58 (0.22)
**All travelers**	401^†^	160	41 (2.4)	22.4	1.78 (0.14)

pdt: person days of travel; SE: standard error. ^†^11 participants travelled to more than one destination. NOTE: Incidence rates were not corrected for the time to first episode of TD or for the number of episodes of TD.

### Travelers' diarrhea: incidence, symptoms, treatment and risk factors

One hundred and sixty travelers (160/390, 41%) (26% per any two weeks of stay) contracted TD. Of these 160 travelers with TD, 16 did not have any accompanying symptom of enteric disease such as nausea, abdominal cramps, vomiting, fever or fecal urgency, making the incidence proportion of classic TD 37% (144/390). The overall TD Incidence Rate (IR) was 1.78 cases per 100 person days of travel (pdt). IRs were highest for travelers to Northern-Africa (3.95/100 pdt) and South-central Asia (2.55/100 pdt) (Table 2). Most affected travelers had typical symptoms: watery stools (138/160, 86%), fecal urgency (114/160, 71%) and abdominal discomfort (123/160, 77%) (Table 3). The diarrheal episode lasted a median of 2.5 days (IQR 1-2.5 days). Sixty-five of 160 travelers with TD (41%) started treatment with an anti-motility agent or an antibiotic: 26% (41/160) used loperamide only, 4% (6/160) activated carbon only, 3% (4/160) used both loperamide and activated carbon and 9% (14/160) used an antibiotic, of whom most (9/14, 64%) used the antibiotic in combination with an anti-motility agent. Five travelers who used an antibiotic (5/14, 36%) had been prescribed the antibiotic at the pre-travel consult. Loperamide was started a median of 1 day (IQR 0-2 days) after onset of symptoms. Antibiotics were started later (median 3 days after onset of symptoms; IQR 2-5 days). In total eight travelers (8/160, 5%) consulted a physician for the diarrheal illness of whom two (2/160; 1%) were admitted to hospital in Africa with fever, diarrhea, vomiting and dehydration. One hundred and two travelers (102/390, 26%) reported non-travelers' diarrhea related health problems: 13 vomiting without diarrhea, 11 abdominal discomfort or loose stools that did not fit the definition of TD, 6 constipation, 24 a respiratory tract infection, 19 a skin or eye infection, 3 a urinary tract infections, 2 fever (1 unknown cause, 1 malaria), 12 headache or tiredness, and 12 some other health problem.

**Table 3:** Characteristics of the episode of travelers' diarrhea for 160 Dutch travelers, stratified by the objective degree of inconvenience.

Objective degree of inconvenience - *n *(%)	Conducted program as planned 107/160 (67%)	Forced to alter program 33/160 (21%)	Confined to accommodation 20/160 (13%)	Total 160 (100%)
**Stool frequency **- *n *(%)				
3 stools/day	64 (60)	11 (33)	1 (5)	76 (48)
4-5 stools/day	35 (33)	15 (46)	8 (40)	58 (36)
6-10 stools/day	7 (7)	6 (18)	9 (45)	22 (14)
> 10 stools/day	1 (1)	1 (3)	2 (10)	4 (3)
**Watery stools, duration **- *n *(%)				
No watery stools	20 (19)	1 (3)	1 (5)	22 (14)
1 day	37 (35)	11 (33)	4 (20)	52 (32)
2-3 days	31 (29)	14 (42)	8 (40)	53 (33)
4-7 days	10 (9)	5 (15)	5 (25)	20 (13)
> 7 days	9 (8)	2 (6)	2 (10)	13 (8)
**Fecal urgency, duration **- *n *(%)				
No fecal urgency	38 (36)	8 (24)	-	46 (29)
1 day	30 (28)	10 (30)	7 (35)	47 (29)
2-3 days	22 (21)	10 (30)	6 (30)	38 (24)
4-7 days	11 (10)	3 (9)	1 (5)	15 (9)
> 7 days	6 (6)	2 (6)	6 (30)	14 (9)
**Abdominal cramps, duration **- *n *(%)^†^				
No abdominal cramps	32 (30)	3 (9)	2 (10)	37 (23)
1 day	32 (30)	11 (33)	4 (20)	47 (29)
2-3 days	30 (28)	12 (36)	6 (30)	48 (30)
4-7 days	9 (8)	5 (15)	4 (20)	18 (11)
> 7 days	4 (4)	2 (6)	4 (20)	10 (6)
**Nausea, duration **- *n *(%)				
No nausea	82 (77)	13 (39)	4 (20)	99 (62)
1 day	17 (16)	9 (27)	6 (30)	32 (20)
2-3 days	6 (6)	8 (24)	6 (30)	20 (13)
4-7 days	1 (1)	2 (6)	3 (15)	6 (4)
> 7 days	1 (1)	1 (3)	1 (5)	3 (2)
**Vomiting **- *n *(%)*	13 (12)	12 (36)	7 (35)	32 (20)
**Fever **- *n *(%)	6 (6)	8 (7)	11 (55)	17 (11)
**Treatment **- *n *(%)				
Loperamide	29 (27)	11 (33)	14 (70)	54 (34)
Activated carbon	3 (3)	6 (18)	2 (10)	11 (7)
Antimicrobial agent	3 (3)	6 (18)	5 (25)	14 (9)
**Subjective degree of inconvenience **- *n *(%)				
None/Minor	58 (54)	5 (15)	-	63 (39)
Moderate	33 (31)	13 (39)	8 (40)	54 (34)
Large/Severe	16 (15)	15 (46)	12 (60)	43 (27)

*13 additional travelers who did not have diarrhea reported vomiting; ^†^10 additional travelers who did not have travelers' diarrhea according to the definition, reported abdominal cramps.

The following variables independently increased the odds of contracting TD: younger age, use of an antacid, longer travel duration, lower Human Development Index of the country that was visited, backpacking as type of travel and staying in luxury hotels. Travelers whose main travel purpose was to visit friends/relatives or who traveled for business/professional reasons had reduced odds for contracting TD. Nagelkerke's R^2 ^was 0.22, which means that the model accounted for 22% of the variance in TD (Table 1).

### Inconvenience due to travelers' diarrhea

Although most travelers (107/160; 67%) could conduct their activity program as planned despite having diarrhea, 21% (33/160) were forced to alter their program and an additional 13% (20/160) were confined to their accommodation for one or more daylight days (median 1 day; IQR 1-2 days). When asked about the degree of inconvenience brought on by the diarrheal illness, 39% (63/160) categorized it as minor or none at all, 34% (54/160) as moderate and 27% (43/160) as large or severe. Severity of symptoms was greater and use of treatment and necessity to alter the activity program were more common in those who were incapacitated due to TD (Table 3). In a logistic regression model, restricted to travelers who contracted TD, none of the person- or travel-characteristics were significantly (*p *< 0.05) associated with incapacitation due to TD. The following symptoms independently increased the odds of incapacitation due to TD: stool frequency, nausea and fever (Table 4).

**Table 4:** Logistic regression model evaluating which symptoms best predicted incapacitation due to travelers' diarrhea.

Characteristic	All with TD n = 160	Conducted program as planned n = 107	Incapacitated n = 53	Univariate OR [95% CI]	*p*-value	Multivariate OR [95% CI]
**Stool frequency**					< 0.001	
3 stools/day	76 (48)	64 (60)	12 (23)	1.0		1.0
4-5 stools/day	58 (36)	35 (33)	23 (43)	3.51 [1.56-7.88]		2.05 [0.77-5.43]
> 5 stools/day	26 (16)	8 (8)	18 (34)	12.0 [4.26-33.8]		4.84 [1.40-16.8]
**Abdominal cramps**					0.005	
No abdominal cramps	37 (23)	32 (30)	5 (9)	1.0		1.0
1 -3 days	95 (59)	62 (58)	33 (62)	3.41 [1.21-9.57]		1.86 [0.55-6.34]
> 3 days	28 (18)	13 (12)	15 (28)	7.39 [2.22-24.5]		2.64 [0.62-11.3]
**Fecal urgency**^1^	114 (71)	69 (65)	45 (85)	3.10 [1.32-7.25]	0.009	0.93 [0.32-2.70]
**Nausea**^2^	61 (38)	25 (23)	36 (68)	6.95 [3.35-14.4]	< 0.001	4.38 [1.70-11.3]
**Vomiting**^3^	32 (20)	13 (12)	19 (36)	4.04 [1.80-9.06]	0.001	0.96 [0.32-2.91]
**Fever**^4^	25 (16)	6 (6)	19 (36)	9.41 [3.47-25.5]	< 0.001	5.65 [1.80-17.7]

TD: travelers' diarrhea; OR: odds ratio; CI: confidence interval. *P*-values based on χ^2^-tests for categorical variables. Variables with *p *< 0.2 were included in the multivariate logistic regression model. Reference category: ^1^no fecal urgency, ^2^no nausea, ^3^no vomiting, ^4^no fever. Model: constant = 0.06, Nagelkerke's R^2 ^= 0.42, Hosmer and Lemeshow test for goodness of fit *p *= 0.7.

Before departure, we surveyed the incidence of diarrhea among participants during a two-month period in The Netherlands; 22% answered that they had an episode of diarrhea according to our definition of (travelers') diarrhea. The normal stool pattern of these participants may come close to fulfilling the definition of TD, making these participants more likely to report TD during travel without significant inconvenience. Therefore, we performed a sensitivity analysis in which we excluded these participants. This did not cause a major change in the results. In the remaining subset 62% could conduct their activity program as planned, 27% were forced to alter their program and 10% were confined to their accommodation. In another sensitivity analysis restricted to 144 participants with classic TD, 65% could conduct their activity program as planned, 22% were forced to alter their program and 14% were confined to their accommodation.

Before departure all travelers were asked the following question: "If you were to contract travelers' diarrhea with fecal urgency and abdominal cramps for three days, how large a problem would you consider this to be?". After returning home all travelers were presented a similar scenario. Table 5 shows that the distribution of participants' answers did not shift in those who did not contract TD (p = 0.6, Wilcoxon signed rank test for two-related samples, comparison of the distribution of two variables). However, those who did contract TD tended to consider TD a smaller problem when asked the question upon return than they had thought it would be prior to departure (p < 0.001). Surprisingly, even the participants who were forced to alter their planned activities (p = 0.01) and the participants who were forced to stay indoors (p = 0.03) tended to consider TD less of a problem when asked the question upon return than they had thought it would be before departure.

**Table 5:** How did an episode of travelers' diarrhea (TD) influence travelers' perception of TD? The expected amount of subjective inconvenience due to travelers'' diarrhea before and after travel is stratified by whether travelers had TD.*

	Travelers who had TD n = 160		Travelers who did not have TD n = 230	
	**Before departure**	**After returning**	**Before departure**	**After returning**
No problem - *n *(%)	1 (1)	11 (7)	1 (0.4)	3 (1)
A small problem - *n *(%)	22 (14)	42 (26)	50 (22)	53 (23)
Neither a small nor a large problem - *n *(%)	51 (32)	56 (35)	61 (27)	57 (25)
A large problem - *n *(%)	69 (43)	49 (31)	99 (43)	99 (43)
A very large problem - *n *(%)	17 (11)	2 (1)	19 (8)	18 (8)

*Participants were presented the following scenarios: *Before departure: *If you were to contract travelers' diarrhea during the coming journey, with a duration of three days accompanied by urgency and abdominal cramps, how large a problem do you think this would be for you? *After returning*: If you were to make the exact same journey in the future and you were to contract travelers' diarrhea with a duration of three days accompanied by urgency and abdominal cramps, how large a problem do you think this would be for you?.

## Discussion

This study was specifically designed to measure the degree of inconvenience brought on by TD. We found that approximately one-third of travelers who contracted TD were forced to change their activity program or stay indoors, which is in line with other reports [2,3,13-15]. Two travelers were even admitted to hospital. Two-thirds did not need to change their activity program and a sizeable proportion (39%) said that the episode of TD caused only minor inconvenience. Those who reported minor inconvenience seldom used an anti-diarrheic agent meaning that the reported degree of inconvenience in this subgroup was not significantly influenced by treatment. As it is to be expected, the severity of symptoms was greater in those who regarded the episode of TD a major inconvenience. The travelers' perception of TD changed based on the current experience. Travelers who contracted TD considered it less of a problem in retrospect than they had thought it would be before departure. Surprisingly, this was even true for those who were forced to change their plans and for those who had to stay indoors. Although this finding may simply mean that travelers are less apprehensive about problems they have faced before, it suggests that TD is less of a nuisance than travelers expect beforehand.

Most risk factors for contracting TD were in line with recent reports. Unexpectedly, we found that staying in luxury hotels increased the odds for contracting TD. Travel duration for participants who stayed in luxury hotels was shorter and they were more likely to have traveled to high risk destinations, such as Indonesia and Egypt (data not shown). Residual confounding due to incomplete adjustment for destination and for the time to the first episode of TD may account for (part of) the unexpected association between accommodation in luxury hotels and TD. Alternatively, staying in luxury hotels may be associated with consumption of more elaborate food which bears more risks [13].

This study has a number of strengths. First, participants were recruited before departure. This way we aimed to limit the chance of preferentially selecting travelers with more severe TD who may be more inclined to respond to a questionnaire taken after the facts. Secondly, surveying travelers both before- and after travel, enabled analysis of how travelers' perception of TD changed depending on whether or not TD was contracted during travel. Thirdly, nearly all participants completed both questionnaires, further limiting the chance of bias. Lastly, we measured both the objective and subjective degree of inconvenience. Participants' reporting of both kinds of inconvenience was consistent, which shows that the data are robust. The study also has limitations. First, although we piloted the questionnaire among travelers, acquaintances and epidemiologists, questions could have been misinterpreted. To limit the chance of misinterpretation, participants could contact us by e-mail in case of any ambiguity. We also provided ample opportunity for participants to further specify answers. For example, those who reported that they had to change their activity program or remain indoors due to TD were requested to describe which activities were cancelled. Secondly, the normal stool pattern of some participants may come close to fulfilling the definition of TD, making these participants more likely to report TD without significant inconvenience. This may have led to an underestimation of the inconvenience associated with 'real TD'. However, two sensitivity analyses in which such participants were excluded did not yield different results. Therefore it is unlikely that we underestimated the inconvenience associated with TD during the stay abroad. Thirdly, many travelers used an anti-motility agent or an antibiotic to treat TD. It stands to reason that the degree of inconvenience would have been larger if nobody had used treatment and would have been smaller if all had used treatment. Lastly, TD incidence rates were not corrected for the time to the first episode of TD or for the number of episodes. This may have inflated incidence rates for destinations for which travel duration was longer than average and deflated incidence rates for destinations for which it was shorter than average.

Most cases of TD in this study fitted the classic definition of TD. Overall incidence rates and risk factors were in line with recent reports [1,3,13,14,23]. These aspects increase the generalizability of this study. Some aspects limit the generalizability. Firstly, the study population consisted mainly of Dutch born nationals. Dutch people may be more inclined to await the natural course of a self-limiting illness than travelers from other countries [24]. This could influence the way in which they perceive TD as a problem. However, such cultural differences would probably not impact the objective degree of inconvenience. Secondly, participants were recruited at our travel clinic. The results may not be representative of travelers who do not seek health-related travel advice before travel. Furthermore, the response rate was 50%. The demographic features of those who refused to participate may be different. Lastly, although the majority of visitors to our hospital based travel clinic can be classified as 'general travelers', relatively more hospital employees and (bio)medical students visit our travel clinic compared with other out-of-hospital based travel clinics.

## Conclusion

This study shows that conventional definitions of TD encompass many cases of mild TD (in our study at least a third of all cases) for which vaccination or antibiotic treatment is unlikely to provide a significant health benefit. By measuring the degree of inconvenience brought on by TD, researchers and policy makers may be able to better distinguish 'significant TD' from mild TD, thus allowing for a more precise estimation of the size of the target population for vaccination or stand-by antibiotic prescription and of the benefit of such measures. We suggest that a future study should investigate to what extent routine stand-by antibiotic prescription impacts on the subjective and objective degree of inconvenience due to TD as well as the incidence of chronic gastro-intestinal complaints. This could be done by randomizing a similar group of travelers at the pre-travel consult, either to receive a stand-by antibiotic prescription or not.

## List of abbreviations

TD: Travelers' diarrhea; UN: United Nations; HDI: Human Development Index.

## Competing interests

The authors declare that they have no competing interests.

## Authors' contributions

DS and JV were involved in the study design, in data collection, in the analysis and interpretation of the data and in drafting the article. LV was involved in the study design, in the interpretation of the data and in drafting the article. All authors gave final approval to the manuscript.

## Pre-publication history

The pre-publication history for this paper can be accessed here:

http://www.biomedcentral.com/1471-2334/11/322/prepub
